# Polarization-Sensitive Patterning of Azopolymer Thin Films Using Multiple Structured Laser Beams

**DOI:** 10.3390/s23010112

**Published:** 2022-12-22

**Authors:** Alexey P. Porfirev, Svetlana N. Khonina, Nikolay A. Ivliev, Sergey A. Fomchenkov, Denis P. Porfirev, Sergey V. Karpeev

**Affiliations:** Image Processing Systems Institute of RAS—Branch of the FSRC “Crystallography and Photonics” RAS, 443001 Samara, Russia

**Keywords:** azopolymer, polarization, cylindrical polarization, laser material processing, diffractive beam splitter

## Abstract

The polarization sensitivity of azopolymers is well known. Therefore, these materials are actively used in many applications of photonics. Recently, the unique possibilities of processing such materials using a structured laser beam were demonstrated, which revealed the key role of the distribution of polarization and the longitudinal component of light in determining the shape of the nano- and microstructures formed on the surfaces of thin azopolymer films. Here, we present numerical and experimental results demonstrating the high polarization sensitivity of thin azopolymer films to the local polarization state of an illuminating structured laser beam consisting of a set of light spots. To form such arrays of spots with a controlled distribution of polarization, different polarization states of laser beams, both homogeneous and locally inhomogeneous, were used. The results obtained show the possibility of implementing a parallel non-uniform patterning of thin azopolymer films depending on the polarization distribution of the illuminating laser beam. We believe that the demonstrated results will not only make it possible to implement the simultaneous detection of local polarization states of complex-shaped light fields but will also be used for the high-performance fabrication of diffractive optical elements and metasurfaces.

## 1. Introduction

The possibility of processing materials not with one laser beam but with multiple laser beams has made it possible to increase the processing speed by orders of magnitude [1,2,3,4,5]. This has resulted in significant cost savings. The two most well-known approaches for the realization of laser material processing with multiple laser beams are interferometric lithography [4] and the use of diffractive beam splitters [1,2,5,6]. These two methods show high efficiency and accuracy. However, the generated light spots in both cases have the same structure. This is beneficial when it is required to form arrays of absolutely identical nano- or microstructures on the processed surface [1,2], but it does not allow the simultaneous fabrication of arrays of structures with different profiles. The latter technique may be required not in the manufacturing of some two-dimensional or one-dimensional gratings or arrays of identical structures but in the manufacturing of complex patterns of diffractive optical elements and multifunctional metasurfaces as well as various filters [7]. To implement this approach, it is possible to use a combination of multiplexed structured laser beams with inhomogeneous polarization states [8,9] and polarization-sensitive materials [10]. The so-called azopolymers, azobenzene-containing polymers, are one of the materials that demonstrate polarization-sensitive mass transfer both at the meso- and macrolevels [11]. The orientation of azobenzene molecules changes when irradiated with polarized light [12,13]. Because of this, we can control the characteristics of the relief formed in azopolymers, such as the contrast of diffraction gratings—in this case, interference recording methods show the greatest efficiency when using two Gaussian beams with orthogonal P:P polarizations compared with laser beams with other combinations of polarization (for example, two orthogonal S:S polarizations or different circular polarizations) [14].

We should note that some polarization transformation and generation of multiple laser beams with different polarization states is possible with diffractive optical elements [8,9,15,16]. This is possible, for example, because of the polarization and phase interaction of non-uniformly polarized laser beams with singular-phase structures [9]. However, there are some limitations, and only several types of polarization transformations are possible. More controllable polarization transformation can be achieved using 4-*f* optical systems with spatial polarization filtering [17]. In this case, a designed polarizing filter is located in the Fourier plane and transforms the polarization state of the input linearly polarized structured laser beam. As such a polarizing filter, a quarter-wave plate or a half-wave plate can be used to identically change the polarization state at every point of the initial beam. More complex polarization transformation is possible with *q*-plates/S-waveplates [18,19], which are, in fact, segmented wave plates with spatially variable retardation [20]. Then, if the element with a phase function of a diffractive beam splitter is located in the input plane of the 4-*f* optical system, we can use such a polarization filter to transform the linear polarization of each multiplexed laser beam into a linear polarization with the desired polarization direction. Such transformed laser beams can be used for locally variant patterning of polarization-sensitive azopolymers films for the fabrication of various elements of diffractive optics and photonics.

In this study, we used thin films of azopolymers based on 9-(2,3-Epoxypropyl)carbazole (EPA) and dispersed orange DO3 azo dye (Disperse Orange 3, DO3) [14,21]. Previously, these materials showed high sensitivity to the polarization of the illuminating structured laser beam and were used for the visualization of the longitudinal component of both uniformly and non-uniformly polarized (including cylindrical and hybrid-cylindrical polarization) laser beams [22]. In contrast to Ref. [22], in which we used *q*-plates for the generation of single cylindrically polarized laser beams, in this work, we used *q*-plates for the generation of a set of linearly polarized laser beams with different orientations of the polarization axis for each beam.

The high polarization sensitivity of this material allowed us to use it as a detector of the local polarization distribution. In addition, the use of non-uniformly polarized light for the processing of this material shows the possibility of the fabrication of ordered sets of microprotusions with predetermined orientations. This can be used for the formation of pre-designed relief patterns in thin azopolymer films for the high-throughput fabrication of diffractive optical elements and metasurfaces.

## 2. Methods

**Figure 1:**
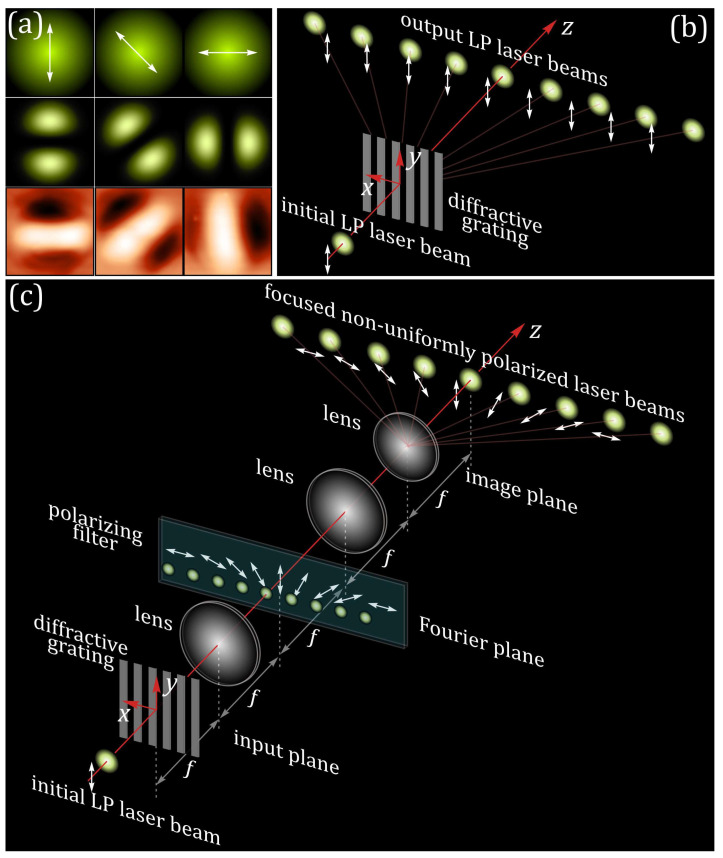
Concept of approach for implementation of polarization-sensitive patterning of azopolymer thin films using multiple structured laser beams. (**a**) Intensity distributions (top row) and the longitudinal components (middle row) of focused linearly polarized (LP) Gaussian laser beams with different polarization directions, as well as images of the microstructures formed in thin azopolymers films under the illumination of these beams (bottom row). (**b**) Splitting of a single LP Gaussian laser beam into a set of LP laser beams with a one-dimensional diffractive grating. (**c**) Principle of spatial polarization filtering and generation of a set of LP laser beams with different polarization directions using a 4-*f* optical system with a polarizing filter.

Previously, it was shown that with laser processing of thin azopolymer films with polarized light, the profile of the fabricated microstructures is proportional to the inverse intensity of the longitudinal component of the illuminating light [22]. For focused linearly polarized light with different polarization directions, the longitudinal components have the profiles presented in Figure 1a. The formed microstructures are protrusions elongated in the direction of the light polarization. Controlling the direction of the linear polarization allows one to control the orientation of the formed protrusions. To speed up laser processing, splitting a single laser beam with diffractive optical elements or metasurfaces can be used—in this case, the polarization structure of each of the generated multiple beams copied the polarization structure of the initial beam (Figure 1b). However, the use of the 4-*f* optical system for spatial filtering allows one to control the polarization state of each of the generated light spots independently (Figure 1c). In conventional 4-*f* optical systems, amplitude/phase or polarization filters are used for the realization of such operations as, for example, edge imaging, contrasting, and the optical implementation of different mathematical operations [23,24,25]. Polarizing filters with locally changeable orientations of the polarization vector direction can be used to encode information on the polarization direction in the input light field. Then, polarization-sensitive materials, such as azopolymers, can be used to decode and visualize this information.

Each light spot shaped at the Fourier plane of the 4-*f* optical system is incident on different areas of the polarizing filter. If a half-wave plate is used as a polarizing filter, each section of the filter performs the same transformation of the initial linear polarization of each light spot—for all spots, the polarization axis rotates by the same angle, equal to the double angle of rotation of the half-wave plate axis. When a *q*-plate is used as a polarizing filter in each region of the plate where the shaped light spots are incident, the angle of the rotation of the original linear polarization is determined by the structure of the *q*-plate in that region. Since *q*-plates are, in fact, a set of half-wave plates, the rotation angles of the linear polarization are different for each shaped spot. Therefore, with *q*-plate generating *m*-order cylindrical polarization, the angle of rotation of the polarization after the plate is defined as *m*ϕ, where ϕ is the polar angle [26,27]. In contrast to Ref. [22], in which *q*-plates were used for single cylindrically polarized laser beam generation, here, we used *q*-plates for the generation of a set of linearly polarized laser beams with different orientations of the polarization axis for each beam. This made it possible to realize parallel non-uniform laser patterning of thin azopolymer films.

To design a diffractive optical element splitting an initial Gaussian beam into a set of light spots arranged in a circle, we used the method of carrier spatial frequencies [28] and a modification of the method of designing a diffractive beam splitter that enables the diffraction orders to be uniformly distributed within a required elliptic region, which was proposed by Skidanov and Kazanskiy [29]. The phase function of this element is defined as a set of diffractive gratings producing the diffractive orders, which are equidistant in the polar coordinates:

(1) $$T\left(r,\phi \right)=\sum_{m=1}^{M}\sum_{n=1}^{N}C_{mn}\exp\left[ikrR_{m}\cos\left(\phi -\upsilon_{mn}\right)\right]$$

where (*r*, *φ*) are the polar coordinates in the initial plane, *k* = 2*π*/*λ* is the wavenumber, *λ* is the wavelength, and (*R_m_*, υ*_mn_*) are the spatial frequencies that determine the positions of the diffraction orders in the Fourier plane. For the experiments, we designed a diffractive optical element splitting an initial linearly polarized Gaussian beam into a set of 24 light spots located on two circles (*M* = 2) with radii *R*_1_ and *R*_2_; the light spots were evenly distributed along the angular coordinate, with 8 light spots on the first circle and 16 light spots on the second circle.

With light field modulation using a 4-*f* optical system with a polarizing filter, the light field polarization state in the Fourier plane undergoes some polarization transformations:

(2) $$C_{out}\left(\upsilon \right)=P\left(\upsilon \right)C_{in}$$

where ***C****_in_* and ***C****_out_* are the input and output polarization vectors, respectively, and $P\left(\upsilon \right)$ is the Jones matrix of the corresponding polarization transformation [7,8,9,17,30]. In particular, in this paper, we consider a linearly polarized input field ***C****_in_* = (*c_x_*, *c_y_*)*^T^* and polarization transformations corresponding to *m*-th order radial polarization [31].

Then, to calculate the components of the focused modulated light field, we used the well-known Debye approximation and Richards–Wolf formulas [32] are:

(3) $$\begin{array}{l} E(\rho ,\psi ,z)=\left(\begin{array}{c} E_{x}(\rho ,\psi ,z)\\ E_{y}(\rho ,\psi ,z)\\ E_{z}(\rho ,\psi ,z) \end{array}\right)=-\frac{if}{\lambda }\times \\ \times \int_{0}^{\Theta }\int_{0}^{2\pi }\left(\begin{array}{ll} \left[1+\cos^{2}v(\cos\theta -1)\right] & \sin v\cos v(\cos\theta -1)\\ \sin v\cos v(\cos\theta -1) & \left[1+\sin^{2}v(\cos\theta -1)\right]\\ -\sin\theta \cos v & -\sin\theta \sin v \end{array}\right)\left(\begin{array}{c} c_{x}(v)\\ c_{y}(v) \end{array}\right)\times \\ \times B(\theta ,v)T(\theta )\exp\left[ik(\rho \sin\theta \cos(v-\psi )+z\cos\theta )\right]\sin\theta \;d\theta \;dv\;, \end{array}$$

where (*ρ*, ψ, *z*) are the cylindrical coordinates in the focal region, (*θ*, *ν*) are the spherical angular coordinates of the focusing system’s output pupil, Θ is the maximum value of the azimuthal angle related to the system’s numerical aperture, *B*(*θ*, *ν*) is the transmission function, *T*(*θ*) = (cos*θ*)^1/2^ is the pupil’s apodization function of aplanatic systems, *f* is the focal length, and ***C***(*ν*) = (*c_x_*(*ν*), *c_y_*(*ν*))*^T^* is the polarization vector after the implementation of the polarization transformation of Equation (2).

The profiles of surface relief formed on the surface of the thin azopolymers films are approximated well by a model proposed by Ambrosio et al., 2012 [33]. However, it was previously shown that the longitudinal component of the illuminating light field, the calculation of which is less expensive in terms of time and computing power, also can be used as a simple model for the prediction of these profiles. Figure 1a shows some examples of the linearly polarized light beams with different polarization directions and the profiles of the microstructures formed on the surface of a thin azopolymer film.

## 3. Optical Experiment

**Figure 2:**
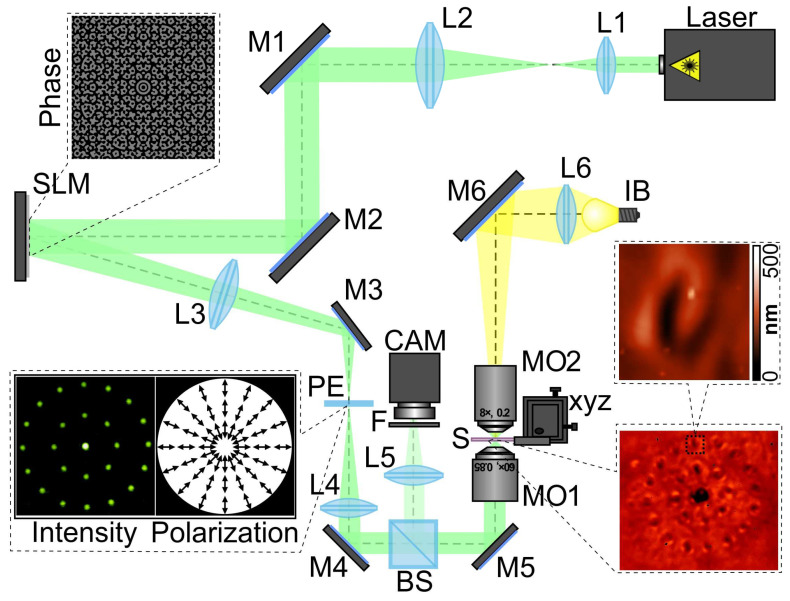
The experimental setup for polarization-sensitive laser patterning of thin azopolymer films. The laser is a solid-state laser (λ=532 nm); L1, L2, L3, L4, L5, and L6 are spherical lenses (*f*_1_ = 350 mm, *f*_2_ = 300 mm, *f*_3_ = 500 mm, *f*_4_ = 400 mm, *f*_5_ = 50 mm, and *f*_6_ = 150 mm); M1, M2, M3, M4, M5, and M6 are mirrors; SLM is a reflective spatial light modulator (HOLOEYE PLUTO VIS); PE is a polarizing element; BS is a beam splitter; MO1 and MO2 are micro-objectives (NA = 0.4 and 0.1); S is a glass substrate with a carbazole-containing azopolymer thin film that was mounted on the 3-axis XYZ translation stage; IB is a light bulb; F is a neutral density filter; and CAM is a ToupCam UCMOS08000KPB video camera. The insets show a phase transmission function of the designed diffractive beam splitter for generation of a set of 24 light spots located on two circles with different radii; intensity and polarization patterns in the Fourier plane of the used 4-*f* optical system; and optical microscopy image of the patterned azopolymer films with an enlarged image of a single fabricated structure obtained using atomic force microscopy.

The optical schema of the experimental setup used for the laser patterning of the thin azopolymer films is shown in Figure 2. The setup was based on the reflective spatial light modulator (SLM) HOLOEYE PLUTO VIS (1920×1080 px, pixel size of 8 μm) for the realization of phase masks of the diffractive optical element performing the splitting of the illuminating Gaussian beam into a set of light spots arranged around a circle. A linearly polarized Gaussian beam from a solid-state laser (λ = 532 nm, *P_out_* = 15 mW) was extended and collimated with a combination of two lenses, L1 and L2, with focal lengths of 25 and 150 mm. The collimated laser beam was directed onto the SLM with the help of mirrors M1 and M2. The 4-*f* optical system, consisting of two lenses, L3 and L4, as well as polarizing element PE, was used for the spatial filtering of the modulated laser beam. For the polarizing element, we used three different elements—a half-wave plate and first- and second-order *q*-plates. The half-wave plate allowed us to control the orientation of linear polarization of the field; the first- and second-order *q*-plates were used to generate polarization patterns corresponding to the cylindrical polarization of the first (azimuthal polarization) and second orders. The polarizing element was located in the focal plane of lens L3. Thus, we had the ability to control the polarization distribution of each formed light spot and obtain different polarization patterns. The spatially filtered laser radiation was directed into the input pupil of a micro-objective, MO1 (NA = 0.40), with the help of mirrors M3, M4, and M5. The glass substrate S with a carbazole-containing azopolymer thin film was mounted on the 3-axis XYZ translation stage and located in the focal plane of micro-objective MO1. The thickness of the azopolymer thin films used was 1.5 µm. A system consisting of a light bulb, IB; a spherical lens, L6 (focal length of 50 mm); a mirror, M6; and a micro-objective, MO2 (NA = 0.1), was used to illuminate the surface of the glass substrate. To observe the surface of the glass substrate with the carbazole-containing azopolymer thin film during laser writing, we used a system consisting of a beam splitter, BS; a lens, L5 (focal length of 150 mm); and a video camera, CAM (TOUPCAM UHCCD00800KPA; 1600 × 1200 px with a pixel size of 3.34 μm). A neutral density filter, F, was used to decrease the intensity of the observed laser beam.

**Figure 3:**
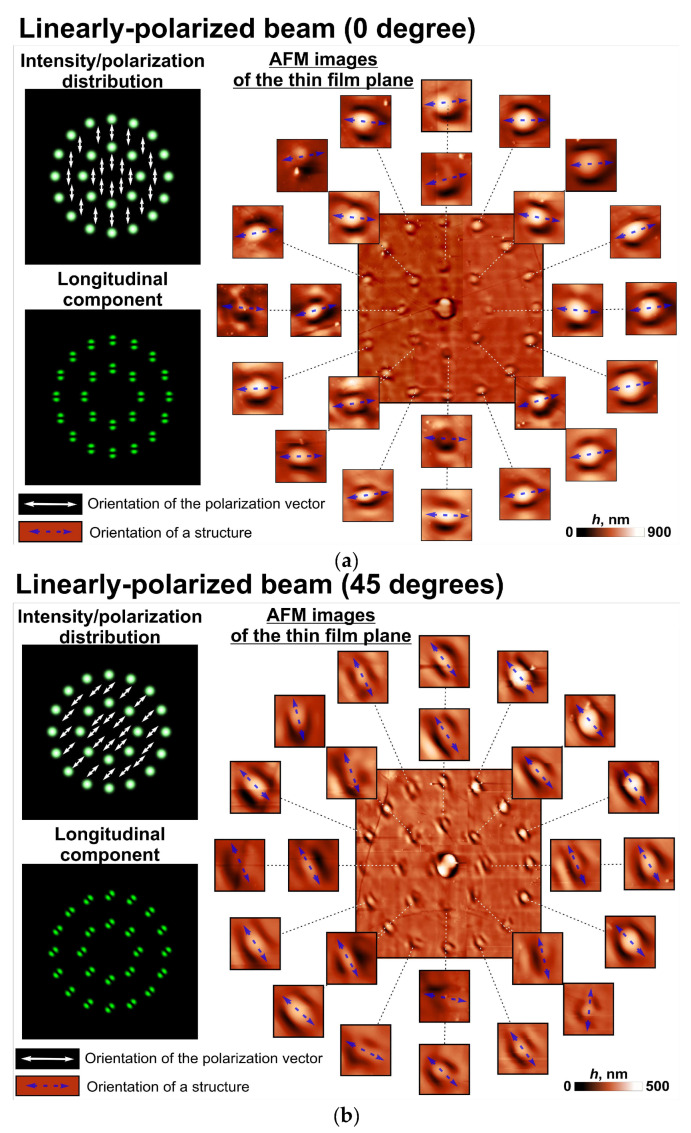
Laser patterning of a thin azopolymer film with a set of modulated linearly polarized spots. Modeling intensity distributions of the used illuminating laser beams as well as atomic force microscopy (AFM) images of the fabricated microstructures for uniform linear polarization with angles of 0 (**a**) and 45 (**b**) degrees are shown.

**Figure 4:**
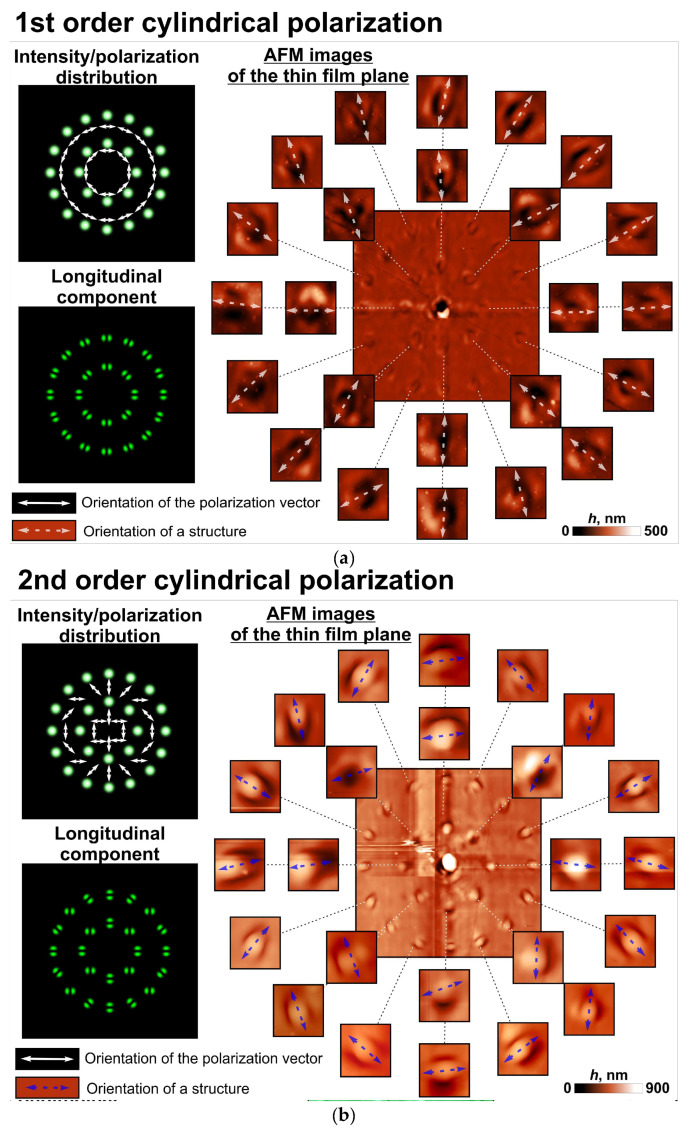
Laser patterning of a thin azopolymer film with a set of modulated polarized spots with non-uniform polarization distributions, which correspond to the first/second order cylindrical polarization. Modeling intensity distributions of the used illuminating laser beams as well as atomic force microscopy (AFM) images of the fabricated microstructures for first-order (azimuthal) cylindrical polarization (**a**), and second-order cylindrical polarization (**b**).

Figure 3 and Figure 4 show the patterns of microstructures that formed on the surface of the thin azopolymer films when we used different polarizing elements as polarization filters in the 4-*f* optical system. These cases include the following polarization patterns: uniform linear polarization with angles of 0 and 45 degrees, first-order cylindrical polarization (azimuthal polarization), and second-order cylindrical polarization. From the analysis of the patterns obtained for the linearly polarized beam, we can conclude that the orientation of each elongated microprotrusion formed was perpendicular to the direction of the local linear polarization at this point. More interesting results were obtained with the first/second order cylindrical polarization pattern—the orientation of the formed microprotrusions replicated the orientations of the polarization vectors of the first/second order cylindrical polarization rotated by 90 degrees. Observed distortion can be explained by the aberration of the optical system used in the experiment, the non-zero dimensions of each light beam in a set, manufacturing errors of the *q*-plates used, and incomplete correspondence of the wavelength of laser radiation (532 nm) to the central working wavelength of the *q*-plate used (515 nm). In fact, two parameters had major effects. The first one was the mentioned incomplete correspondence of the wavelength of laser radiation to the central working wavelength of the *q*-plate used. This led to an incomplete transformation of the initial linear polarization for each light beam in a set and small deviations between the orientations of the polarization vectors and the structures. The second one was the non-zero diffraction-limited dimensions of the generated light spots in a set at the Fourier plane of the used 4-*f* optical system. In this case, part of the used polarization filter illuminated by each light beam in a set performed a non-uniform transformation of the initial linear polarization. This led to the thickening of the formed microprotrusions. In addition, some nanoprotrusions on the film surface also affected the orientation of the formed structures. These nanoprotrusions can also affect the profiles and depth of the formed microprotrusions. Therefore, it is necessary to improve the thin film preparation procedure.

Thus, azopolymer thin films show the possibility to detect not only the whole polarization state of the laser beam, which was shown previously, but also the local modifications of this state. It should be noted that, in this case, the formed relief profiles adequately approximated the longitudinal component of the illuminating beams shown in Figure 3 and Figure 4. This again confirms the assumption about the possibility of using azopolymer thin films to visualize the longitudinal component, which we made earlier, and the possibility of using the longitudinal component of a light field to predict the shape of the formed nano- and microreliefs.

## 4. Discussion

The results obtained show an interesting possibility for the realization of the parallel non-uniform patterning of azopolymer thin films with structured light. Previous demonstrations of the parallel fabrication of multiple nano- and microstructures in azopolymer thin films have been based on the use of interferometric lithography, which does not allow one to control the profile and orientation of each of the formed structures [34,35,36]. Despite this, the interferometric approach has many advantages, among which are the high processing speed and the possibility of the parallel processing of large areas of materials. However, the opportunities provided by the method described in our paper allow one to realize more complex types of parallel high-throughput laser processing of azopolymers. The demonstrated technique is similar to the laser processing of metals with the formation of laser-induced periodic surface structures (LIPSSs) [37]. As with LIPSSs, the orientation of the formed structures is controlled by the local polarization state of the illuminating laser beam, which allows one to use pre-designed complex vector beams with non-uniform polarization states for the fabrication of surface structures with unconventional patterns and shapes [38,39]. Thus, the proposed approach can be considered for direct laser printing [40] in films of photosensitive materials, which has been successfully used to create diffractive optical elements (microlenses, gratings, and fork-gratings) [41,42,43,44] and microelements for chemo- and biosensing [45,46] and may be promising for security label generation [47].

The next important step in the improvement of the proposed technique is the investigation of the effect of the decreasing distances between the formed light spots with different polarization states. The minimization of the effects of the interference between the closely packed light spots allows one to fabricate various diffractive optical elements and metasurfaces with high efficiency and minimal fabrication errors. In this case, as shown in this study and our previous studies, the control of the longitudinal component of the multiple generated light spots plays a vital role.

## 5. Conclusions

An approach for the parallel fabrication of multiple microprotrusions with controlled orientation in azopolymer thin films was developed. The possibility of the control of the profile of each formed microprotrusion was shown experimentally and confirmed with atomic microscopy.

Different polarization patterns, including linear polarization with two different polarization angles, the first- and second-order cylindrical polarization, were used to demonstrate and fabricate multiple microstructures located on two circles with different radii. Laser-written patterns on azopolymer films adequately reproduced the local changes in the polarization of the used structured laser beams in all cases.

The calculated distributions of the longitudinal components of the structured laser beams used in the experiments were in good agreement with the profiles of the fabricated microstructures, which, once again, confirmed the possibility of using the longitudinal component of light to predict the shape of the microstructures formed in the thin azopolymer films. This also allows one to use azopolymer thin films as detectors of the polarization state of the illuminating laser beam—not only the entire beam as a whole but also its individual sections.

Thus, the developed method provides the possibility to use pre-designed structured laser beams for high-throughput complex laser material processing. The method can be used for the fabrication of complex patterns of microprotrusions and the realization of complex-shaped diffractive gratings, diffractive optical elements, and metasurfaces.
